# Acute pancreatitis with abdominal bloating and distension, normal lipase and amylase: A case report

**DOI:** 10.1097/MD.0000000000015138

**Published:** 2019-04-12

**Authors:** Yuan-Yu Wang, Zhen-Yuan Qian, Wei-Wei Jin, Ke Chen, Xiao-Dong Xu, Yi-Ping Mou, Wei Zhang

**Affiliations:** Departments of Gastrointestinal and Pancreatic Surgery, Zhejiang Provincial People's Hospital, People's Hospital of Hangzhou Medical College, Hangzhou, PR China and Key Laboratory of Gastroenterology of Zhejiang Province, Hangzhou, PR China.

**Keywords:** abdominal bloating, acute pancreatitis, amylase, distension, lipase

## Abstract

**Rationale:**

Acute pancreatitis is an inflammatory disorder of the pancreas, and its correct diagnosis is an area of interest for clinicians. In accordance with the revised Atlanta classification, acute pancreatitis can be diagnosed if at least 2 of the following 3 criteria are fulfilled: abdominal pain; serum lipase (or amylase) activity at least 3 times the upper limit of normal; or characteristic findings of acute pancreatitis on contrast-enhanced computed tomography (CT) or, less often, magnetic resonance imaging or transabdominal ultrasonography. Diagnostic imaging is essential in patients with no or slight enzyme elevation. If enzymes are normal in cases with abdominal distension, there is clinical doubt about the diagnosis of acute pancreatitis, so an early CT scan should be obtained and other life-threatening disorders excluded.

**Patient concerns:**

A 50-year-old male presented with a 1-day history of abdominal bloating and distension. On physical examination, abdominal bulging and mild epigastric tenderness were detected. Laboratory evaluation showed normal amylase and lipase. There was no abnormality on abdominal ultrasound or CT of the abdomen and pelvis. On the fourth day of admission, CT of the abdomen and pelvis showed a hypodense lesion in the pancreas surrounded by a moderate amount of peripancreatic fluid.

**Diagnoses:**

In accordance with the revised Atlanta classification, acute pancreatitis was diagnosed, based on the presence of abdominal pain, and the results of the CT scan of the abdomen and pelvis.

**Interventions:**

The patient was treated with fasting, gastrointestinal decompression bowel rest, intravenous rehydration, and somatostatin.

**Outcomes:**

After 2 days of treatment, his abdominal distension was significantly relieved, and the patient was discharged on the seventh day of admission. At the 3-month follow-up, the patient had no recurrence of pancreatitis.

**Lessons:**

This case of abdominal distension could not be explained by common causes, such as ascites, bowel edema, hematoma, bowel distension, or ileus, which led us to suspect pancreatitis.

## 1 Introduction

Acute pancreatitis, an inflammatory disorder of the pancreas, is the leading cause of admission to hospital for gastrointestinal disorders in the United States and many other countries.^[1]^ According to the 2012 Revised Atlanta Classification, the diagnosis acute pancreatitis requires at least 2 of the 3 following criteria:abdominal pain consistent with pancreatitis, serum amylase and/or lipase of at least 3 times the upper limit of the normal value or findings consistent with acute pancreatitis on imaging [contrast-enhanced CT (CECT), MRI or ultrasound].^[2]^ We present a unique case of acute pancreatitis with abdominal distension, normal lipase and amylase, and no findings consistent with acute pancreatitis on imaging [contrast-enhanced CT (CECT), MRI or ultrasound]. With early diagnosis and supportive treatment, the patient recovered uneventfully.

## 2 Case report

**Figure 1:**
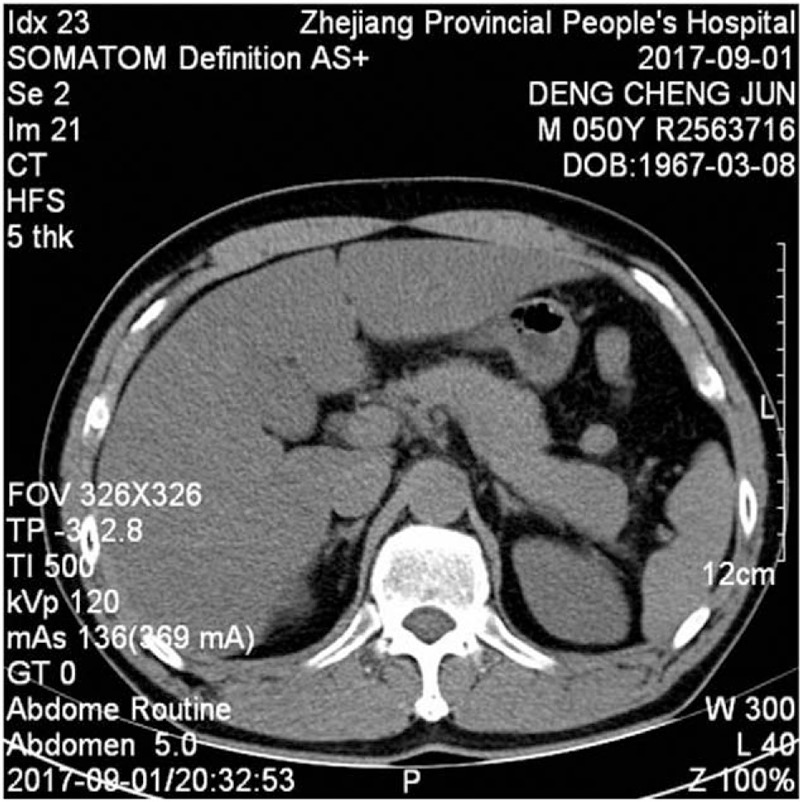
Abdominal computed tomography, with no fluid in the abdominal cavity, no swelling in the pancreas and no expansion of the stomach.

**Figure 2:**
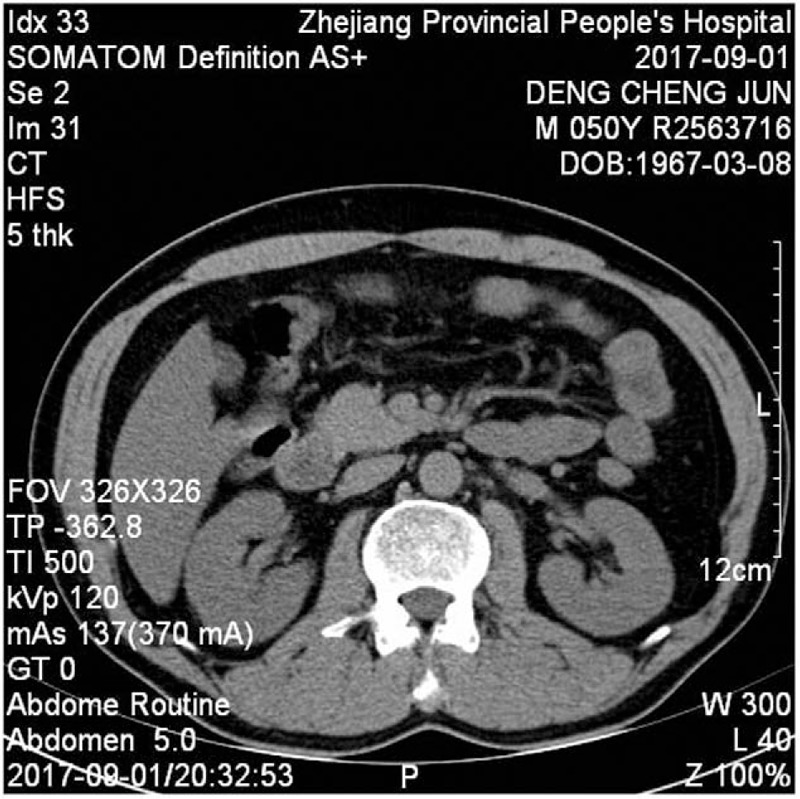
Abdominal computed tomography, with no fluid in the abdominal cavity, no swelling in the pancreas, and no bowel edema, hematoma, bowel distension, or ileus.

**Figure 3:**
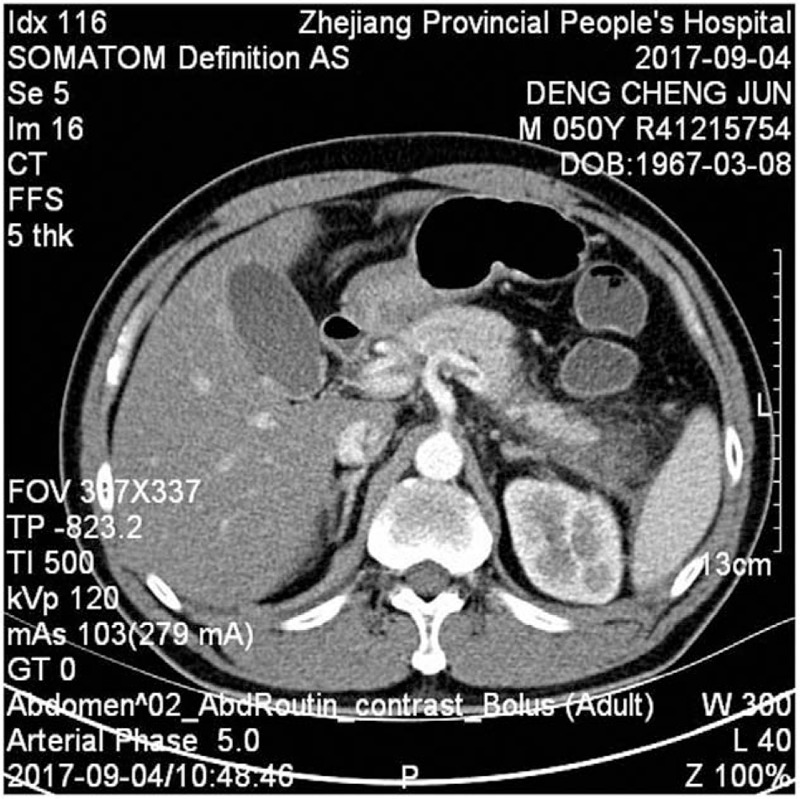
Abdominal computed tomography showing a hypodense lesion in the pancreas surrounded by a moderate amount of peripancreatic fluid.

A 50-year-old male presented with a 1-day history of abdominal bloating and distension, without nausea, vomiting, or diarrhea, and with normal defecation. He denied any history of diabetes or prediabetes, obesity, abdominal trauma, gallstones, and any surgical history. On physical examination, abdominal bulging and mild epigastric tenderness was detected. On the first day of admission, laboratory evaluation showed a normal hematocrit, amylase, lipase, glycerin triglyceride, total protein, and total bilirubin (Table 1). Urinalysis and chest x-ray were unremarkable. Abdominal ultrasound showed a normal gallbladder and liver with normal intrahepatic and extrahepatic bile ducts. Computed tomography (CT) of the abdomen and pelvis showed no fluid in the abdominal cavity, no swelling in the pancreas, and no bowel edema, hematoma, bowel distension, or ileus (Figs. 1 and 2). We therefore diagnosed pancreatitis. The patient was treated with fasting, gastrointestinal decompression bowel rest, intravenous rehydration, and somatostatin. After 2 days of treatment, abdominal distension was significantly relieved. On the fourth day of admission, laboratory evaluation showed that leukocytes and neutrophils were restored to normal, and amylase, lipase, and liver findings were also normal (Table 1). Computed tomography (CT) of the abdomen and pelvis showed a hypodense lesion in the pancreas surrounded by a moderate amount of peripancreatic fluid (Fig. 3). The patient was discharged on the seventh day of admission. At the 3-month follow-up, the patient had no recurrence of pancreatitis. The study was approved and monitored by the ethics committee of Zhejiang Provincial People's Hospital (KT2017030).

**Table 1:**
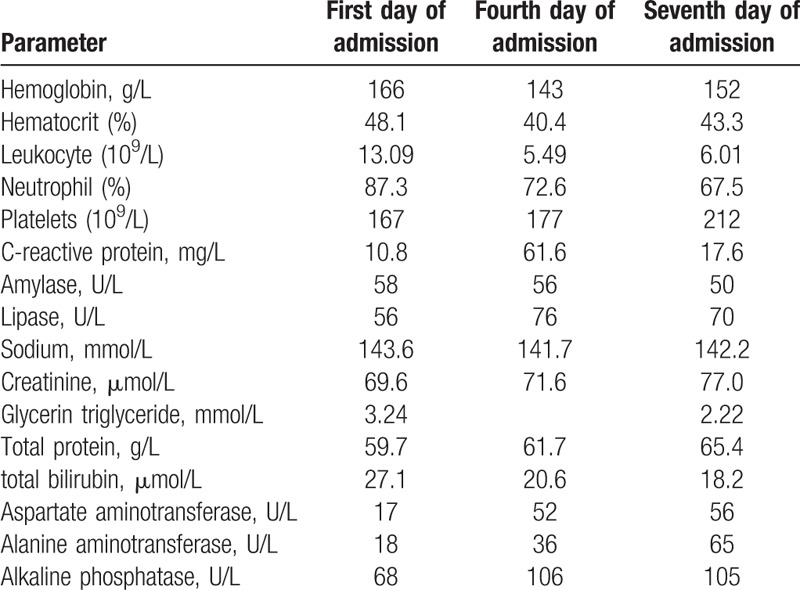
Hematological and biochemical investigations.

## 3 Discussion

Bloating as a symptom and abdominal distension as a sign, are both common functional-type complaints and are challenging to manage effectively. Causes of abdominal distension are related to an increase in intra-abdominal volume: ascites, bowel edema, hematoma, bowel distension, or ileus.^[3]^ Bloating and distension may produce significant distress.^[4,5]^ Bloating and abdominal distension may be produced by different mechanisms, sometimes coinciding in the same individual.^[6]^ Recognizing the predominant operative mechanism(s) in a given patient can help plan effective treatment. To avoid mishaps, organic bloating and distension should always be considered first and appropriately assessed. A mechanism-based management of bloating and distension is ideal but elucidating key operational mechanisms in individual patients is not always feasible. Some clues may be gathered through a detailed dietary history, by assessing bowel movement frequency and stool consistency, and special imaging techniques to measure abdominal shape during episodes of distension. In severe, protracted cases it may be appropriate to refer the patient to a specialized center where motility, visceral sensitivity, and abdominal muscle activity in response to intraluminal stimuli may be measured. In this case, there was no nausea, vomiting, or diarrhea, defecation and vent was normal, computed tomography (CT) of the abdomen and pelvis showed no fluid in the abdominal cavity, no swelling in the pancreas, no bowel edema, no hematoma, bowel distension, or ileus. The abdominal distension of this patient could not be explained by common causes, leading us to suspect pancreatitis.

The annual incidence of acute pancreatitis ranges from 13 to 45 per 100,000 people,^[7]^ and the incidence is rising globally.^[8]^ Acute pancreatitis is among the most common gastrointestinal conditions requiring acute hospitalization.^[9]^ Gallstones and/or biliary sludge are the most prevalent (approximately 40%–50%) cause of acute pancreatitis, alcohol (approximately 20%) is the second most frequent cause of acute pancreatitis, while less frequent causes of acute pancreatitis include medication, endoscopic retrograde cholangiopancreatography, hypercalcemia, hypertriglyceridemia, surgery, and trauma.^[10,11]^

Clinicians are interested in confirming the diagnosis of acute pancreatitis and excluding differential diagnoses. In accordance with the revised Atlanta classification, acute pancreatitis can be diagnosed if at least 2 of the following 3 criteria are fulfilled: abdominal pain (acute onset of persistent and severe epigastric pain, often radiating to the back); serum lipase (or amylase) activity at least 3 times the upper limit of normal; or characteristic findings of acute pancreatitis on contrast-enhanced CT or, less often, MRI or transabdominal ultrasonography.^[2]^ Importantly, pancreatic enzyme concentrations on admission are not associated with disease severity.^[12]^ The disease can be serious, even fatal, although the enzymes are only slightly increased (<3-times normal), so diagnostic imaging is essential in patients with a no or slight enzyme elevation. Enzymes were normal in this case with abdominal distension, introducing clinical doubt about the diagnosis of acute pancreatitis. An early CT scan should be obtained in such cases, and other life-threatening disorders excluded. Computed tomography (CT) of the abdomen and pelvis showed no fluid in the abdominal cavity, and no swelling in the pancreas and no expansion of gastrointestinal tract in this case.

Acute pancreatitis causes a local and systemic inflammatory response syndrome. Although the majority of patients have a mild disease course, around 20% will develop moderate or severe pancreatitis, with necrosis of the (peri)pancreatic tissue and/or (multiple-)organ failure.^[13]^ Patients with mild acute pancreatitis (no organ failure or systemic or local complications) usually do not need pancreatic imaging and are frequently discharged within 3–7 days of onset of illness.^[1]^ Supportive care with the use of intravenous fluid hydration is a mainstay of treatment for acute pancreatitis in the first 12–24 hours. Early fluid resuscitation is required to correct intravascular depletion in order to reduce morbidity and mortality associated with acute pancreatitis.^[14]^ This patient was discharged on the seventh day of admission. At the 3-month follow-up, the patient had no recurrence of pancreatitis. There are no similar case reports/studies of which we are aware.

## 4 Conclusion

Bloating and abdominal distension are both common functional-type complaints, which produce significant distress and are challenging to manage effectively. When abdominal distension cannot be explained by common causes, such as ascites, bowel edema, hematoma, bowel distension, or ileus, we suspected pancreatitis. This case is a retrospective study and the data may not be comprehensive.

## Acknowledgments

The authors thank Marion Barnett, BSc, from Liwen Bianji, Edanz Editing China (www.liwenbianji.cn/ac), for editing the English text of a draft of this manuscript.

## Author contributions

**Conceptualization:** Yuan-Yu Wang, Wei-wei Jin, Yi-Ping Mou.

**Data curation:** Zhen-Yuan Qian, Wei-Wei Jin, Ke Chen.

**Investigation:** Xiao-Dong Xu

**Methodology:** Xiao-Dong Xu,

**Project administration:** Yuan-Yu Wang, Wei Zhang

**Supervision:** Xiao-Dong Xu.

**Writing – Original Draft:** Yuan-Yu Wang.

**Writing – Review & Editing:** Yuan-Yu Wang, Yi-Ping Mou, Wei Zhang.
